# Evolution and structural resilience assessment of the frozen meat trade network

**DOI:** 10.1371/journal.pone.0342003

**Published:** 2026-02-27

**Authors:** Ming Zhang, Yihua Li, Xiangyu Huang, Ying Fu, Wujun Tian

**Affiliations:** 1 College of Economics and Management, Central South University of Forestry and Technology, Changsha, Hunan, China; 2 Hunan Key Laboratory of Intelligent Logistics Technology, Changsha, Hunan, China; 3 College of Computer Science and Mathematics, Central South University of Forestry and Technology, Changsha, Hunan, China; Yantai Institute of Technology, CHINA

## Abstract

This study aims to reveal the spatiotemporal evolution patterns of the global frozen meat trade network from 2003 to 2023 and assess both its static and dynamic structural resilience. Based on UN commodity trade data, directed weighted networks for beef, pork, mutton, and poultry are constructed. By combining complex network theory and simulation models, the static resilience is analyzed from four dimensions: transmissibility, clustering, hierarchy, and assortativity. The impact of single node failures on network performance is simulated to assess dynamic resilience. The results indicate that the global frozen meat trade network shows a trend of polarization, with China emerging as the largest import hub and Brazil becoming the dominant exporter of beef and poultry, reflecting the coexistence of regionalization and globalization. The static resilience analysis reveals that the weighted network exhibits prominent hierarchy and increased assortativity. The poultry network has the highest transmissibility, while the beef network demonstrates the strongest resilience. Interruption simulation results show that the failure of core nodes causes more significant damage to the weighted network, but the network’s overall invulnerability to disruptions has increased over time. The conclusion emphasizes that the resilience of the frozen meat trade network is driven by both weights and topological structure. Reducing dependence on key nodes through diversified trade partners and optimizing regional cooperation is crucial for enhancing trade stability. The weighted directed network model and dynamic resilience evaluation framework proposed in this study provide new methods for global food trade risk management and supply chain optimization.

## 1. Introduction

With global economic growth and the improvement of living standards, international frozen meat trade has become an important part of global trade. It balances meat supply and demand, promotes economic development, and meets the diversified needs of consumers [1]. According to data from UN commodity trade, the total value of frozen meat trade has increased by 231.6% over the past 20 years, with an average annual growth rate of 11.5%; the trade volume has increased by 78.3%, with an average annual growth rate of 3.9%. Beef, pork, mutton, and poultry are the dominant categories, with average annual growth rates of 3.9%, 4.7%, 2.1%, and 3.4%, respectively. This demonstrates the vitality of frozen meat trade and the differentiated growth of various types of meat. However, the global meat trade landscape is complex and influenced by various factors. To protect domestic industries, many governments adopt policy measures to restrict meat imports and exports [2]. In recent years, public health crises such as the COVID-19 pandemic [3]and African swine fever have posed challenges to the frozen meat supply chain. To fill supply-demand gaps and mitigate the impact of international market shocks, countries need to carefully consider moderately increasing meat imports [4]. Scholars have studied the relationship between meat and the economy: Cheng et al. found that China’s meat imports were negatively correlated with prices and positively correlated with real GDP [5]; Aranda et al. analyzed the import barriers faced by Brazilian chicken [6]; Funk et al. explored the impact of COVID-19 on wild meat trade in Nigerian markets [7]; Calvia pointed out that the global meat price cycle is approximately 3.8 to 4.6 years, with price synchronization intensifying during the COVID-19 pandemic and the Russia-Ukraine conflict. Research on the resilience of the global frozen meat trade network can provide valuable insights for addressing future trade risks [8].

In the field of food studies, numerous research efforts have been devoted to trade network patterns. Ercsey-Ravasz et al. found that international agricultural and food trade networks are highly heterogeneous, with a small number of countries occupying key positions [9]. Torreggiani et al. revealed that international food trade networks have a multi-layered structure, where trade agreements and geographical proximity are critical factors [10]. Chung et al. examined the spatiotemporal dynamics of the global meat trade network, focusing on influences such as trade agreements [1]. Karakoc and Konar discovered a synergistic relationship between the efficiency and resilience of food trade networks when considering weighted factors [11]. Wang and Dai and Wang et al. studied the evolution of global food/agricultural trade networks, noting increasing complexity and structural multi-polarity [12,13]. Clemente et al. found that agricultural and food trade networks are influenced by multiple factors [14]. Silvestrini et al. showed that the strength and complexity of food trade networks have significantly increased [15]. Alhussam et al. found that the centrality of the food trade network is positively correlated with food security, while geographical distance negatively impacts food security [16].

In recent years, research on network resilience has grown significantly. The concept originates from mechanics, where it is used to describe the ability of materials to recover [17], and was later introduced to ecosystem restoration studies [18], eventually expanding to complex social-ecological systems. As complex network theory evolved, academic discussions on network resilience increased, covering fields such as urban networks [19], transportation networks [20], economic networks [21], supply chain networks [22], and ecological networks [23]. Network resilience refers to the ability of network members to collaborate across multiple dimensions, effectively responding to external shocks and pressures, while also possessing recovery or transformation capabilities [24].

In the early stages of the development of complex network theory, researchers focused on the basic topological properties of networks, such as node degree distribution, clustering coefficient, and path length. These characteristics laid the foundation for subsequent research on network resilience. Ash and Newth proposed optimizing network structures to enhance resilience [25]. As the theory advanced, researchers began exploring the application of network resilience in different fields, particularly in supply chains and infrastructure networks. Berche et al. emphasized the importance of key nodes [26], while Reggiani explored the resilience of transportation networks, noting that scale-free networks performed better under random failures. Mid-stage scholars delved deeper into the analysis of network characteristics [27], with Zhao et al. examining the impact of topological structures on supply chain resilience [28]. In recent years, research has expanded into multiple dimensions and fields. Chopra et al. proposed a resilience assessment framework for infrastructure [29], while Wang et al. and Yang et al. analyzed the relationship between network characteristics and resilience [30,31]. Li et al. found that network characteristics better reflect resilience than network type [32]. Wang and Dai explored the application of robustness, elasticity, and centrality indicators [12], while Wang et al. revealed that the complexity of trade relations enhances resilience [33]. Tan and Ma observed that a “two-core separation” structure reduces resilience [34], and Liu et al. analyzed the robustness and resilience conceptual frameworks of international trade networks [35]. Jiao assessed the structural resilience of trade networks [36], while Li et al. analyzed the stability and vulnerability of the global chromium ore trade network [37]. These studies have enriched our understanding of the static resilience of networks.

Research on network resilience in the food sector has also made significant progress. Dolfing et al. analyzed the impact of network topology, climate change, and sudden shocks on the evolution of food trade networks, finding that topology has a significant effect on resource distribution and population growth [38]. Hedlund et al. studied the impact of climate change on the stability of crop trade networks, noting that the maize trade network is the most vulnerable, while the wheat and rice networks are more resilient [39]. Enns et al. explored the increased vulnerability of wildlife meat trade networks during the COVID-19 pandemic [40]. Dong et al. simulated trade node attacks to analyze the resilience of the cold-chain meat product trade network, finding that it demonstrated robustness under random attacks and vulnerability under targeted attacks [41]. Ji et al. and H. Xu et al. studied the structural changes, dynamic evolution characteristics, and potential vulnerabilities of the global food trade network, revealing the tightness of network connections and the resilience differences among different crop trade networks [42,43]. Through a systematic literature review and analysis, this study summarizes and organizes the various indicators related to network structure resilience, as shown in Table 1 below.

**Table 1:** Literature on indicators related to network structure resilience.

Evaluation Dimensions	Measurement Indicators	Some of the Mentioned Literatures
redundancy	network density	Gao et al. [44] (An Editorial Expression of Concern has been issued for this article by Nature due to questions raised about the reproducibility of the key numerical findings. Readers are advised to interpret the conclusions with caution.); Wu et al. [45].
	Number of nodes and edges	Yuan et al. [46]; Chen and Chen [47]; Xu and Xu [48].
	average degree	Kim et al. [49].
connectivity	global efficiency	Bai et al. [50]; Ji et al. [42]; Li et al. [51].
	average path length	Gao et al. [52]; Kim et al. [53]; Herrera et al. [54].
	diameter	Berche et al. [26]; Zhao et al. [28]; Miao et al. [55].
	proportion of nodes in the largest connected subgraph	Kim et al. [53]; Reggiani et al. [27]; Dong et al. [56].
	average number of independent paths	Li et al. [51]; Wu et al. [45].
clustering	clustering coefficient	Wan et al. [57]; Artime et al. [58]; Liu et al. [35].
	Modularity	Chopra et al. [29]; Ash and Newth [25].
	reciprocity	Miao et al. [55].
hierarchy	degree distribution	Artime et al. [58]; Reggiani et al. [27].
	Gini coefficient	Sun et al. [59].
assortativity	Pearson correlation-based assortativity coefficient	Ash and Newth [25]; Sun et al. [59].
cohesion	K-shell	Wu et al. [45]; Wang an Dai et al. [12]
centrality	degree centrality	Yuan et al. [46]; Miao et al. [55]; Meng et al. [60].
	eigenvector centrality	Meng et al. [60]; Ji et al. [42]
	closeness centrality	Clark et al. [61]; Berche et al. [26]; Li et al. [32].
	betweenness centrality	Xu and Xu et al. [48]; Kim et al. [49].
	Pagerank centrality	Meng et al. [60]; Ji et al. [42].

The existing research on trade network patterns and network resilience based on complex network theory provides a valuable foundation for this study.However there are still several research gaps: (1) There is a lack of systematic and widely accepted evaluation standards for trade network resilience. Given the uniqueness of each network, resilience evaluation indicators should be customized for specific networks. (2) In the resilience assessment of the food trade network, although relevant results have been achieved in the research on static resilience related to network structural characteristics, most resilience studies do not take into account the weighted factors such as trade intensity. Research on the resilience of weighted networks is relatively scarce. Meanwhile, research on dynamic resilience assessment under disruptions is still in its infancy. (3) Compared to resilience studies in energy and mineral trade networks, research on food trade networks is relatively insufficient, and there is a significant gap in the resilience studies of frozen meat trade networks. This necessitates a comprehensive analysis of the evolution of frozen meat trade network resilience from both temporal and spatial dimensions.

This study first constructs a directed weighted trade network based on global frozen meat trade data from 2003 to 2023 provided by the United Nations Commodity Trade Statistics Database. Then, by analyzing the network’s structural characteristics and simulating the performance under single node disruptions, it empirically studies the network’s static resilience and dynamic structural resilience. The specific contributions are as follows: (1) From both temporal and spatial perspectives, the static and dynamic resilience of the frozen meat trade network are analyzed and visualized in depth. This analysis highlights the critical role of visualization techniques in studying network resilience. Additionally, this analysis has profound significance for improving the security and stability of frozen meat trade supply chain. (2) A dynamic resilience evaluation model under disruption disturbances is designed, which provides an important supplement and reference to the trade network resilience evaluation framework. (3) By simulating resilience loss caused by single node disruptions, we rank the importance of nodes in the frozen meat trade network and identify key nodes. This research provides a scientific basis for enhancing trade security and stability.

The remainder of this paper is organized as follows: Section 2 introduces the research framework, model construction, and data sources; Section 3 presents the computational results; and the paper concludes with a summary.

## 2. Research methods and data sources

### 2.1. Research framework

**Fig 1:**
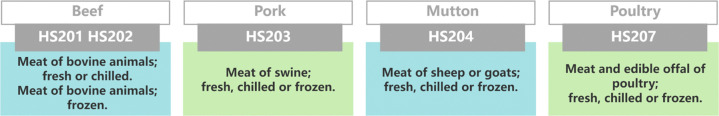
Classification of frozen meat.

This study focuses on frozen meat as the subject of research. Based on existing literature on the definition of frozen meat, it is primarily categorized into four major groups: beef, pork, mutton, and poultry. Although other types of frozen meat are not discussed in this paper, this classification is sufficient to allow for an in-depth analysis of the changes in the global trade network pattern of these four major categories of meat products and their resilience characteristics. The specific classification and corresponding HS codes are shown in Fig 1.

Based on complex network theory and resilience theory, this paper measures the resilience of the global frozen meat trade network from two perspectives: static structural resilience and dynamic structural resilience. Static structural resilience reveals the redundancy and stability of the network from the perspective of its topology, while dynamic structural resilience highlights the network’s vulnerability and invulnerability in response to shocks as it evolves over time. As a typical directed weighted network, the resilience measurement of the global frozen meat trade network requires not only attention to trade relations between countries (regions) but also a focus on the significant impact of trade volume on resilience measurement. Based on existing research and the practical situation of the global frozen meat trade, we have constructed a network structural resilience evaluation indicator system, as shown in Table 2.

**Table 2:** Evaluation indicator system for the structural resilience of the global frozen meat trade network.

Aspect	Influence Factor	Indicator	Impact on the Network
Static structural resilience	transmissibility	global efficiency	Global efficiency measures the speed and capacity of information transmission in the trade network. The higher the efficiency, the smoother the flow of information or materials, resulting in stronger resilience.
	clustering	average clustering coefficient	The average clustering coefficient measures the local structural density of the trade network. The higher the coefficient, the tighter the local clustering between countries (regions), which helps improve the network’s local stability and robustness, as well as its connectivity and transmission efficiency, thereby enhancing network resilience.
	hierarchy	degree distribution	Degree distribution refers to the probability distribution of node degrees. Its impact on network resilience is dual: highly hierarchical structures can provide robustness but also lead to vulnerability; moderate hierarchical and flat structures help strike a balance between the two, improving network resilience.
	assortativity	assortativity coefficient	Assortativity coefficient reflects the degree to which countries (regions) tend to connect with partners that have similar trade volumes. The assortativity coefficient affects the network’s attack resistance ability and stability. Assortative networks strengthen hub connections, providing stability and rapid recovery, while disassortative networks promote information exchange and resource sharing. Due to the different attributes of connected nodes, they offer more redundant paths and recovery capacity.
Dynamic structural resilience	vulnerability	loss rate of network performance caused by single node interruption	Loss rate of network performance caused by single node interruption refers to the percentage decrease in network performance when a single node fails. It reflects the network’s sensitivity to single node failures. A failure of a single node may cause a significant decline in the performance of the entire network, thus measuring the network’s vulnerability and instability.
	invulnerability	average retention rate of network performance	The average performance retention rate of the network refers to the proportion of original performance that the network can retain when facing failures or attacks, measuring the network’s resilience and invulnerability. Networks with a high average performance retention rate are able to quickly recover and retain most of their original performance when facing failures or attacks. This helps ensure the continuous stable operation.

**Fig 2:**
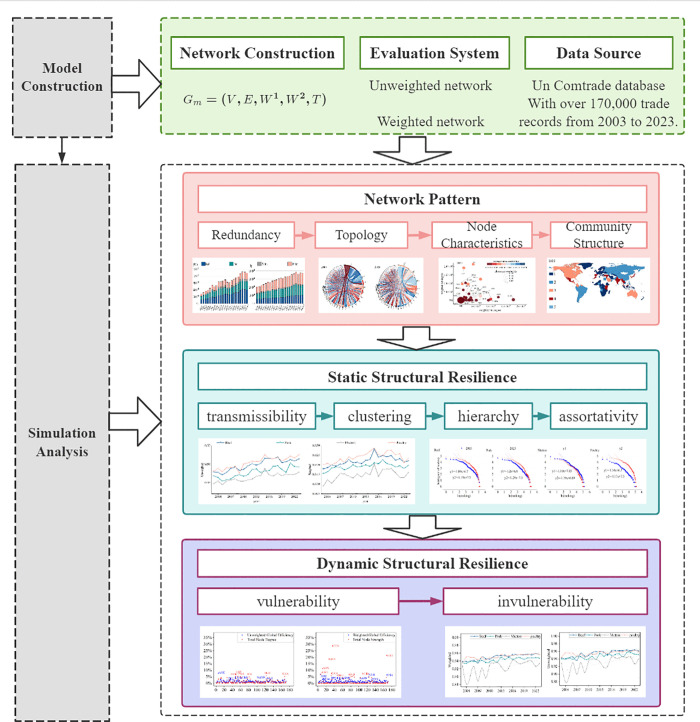
Research framework.

Based on the construction of the above indicator system, further data collection was conducted to establish a global frozen meat directed trade network model, systematically conducting empirical research on network patterns and structural resilience. The research framework is shown in Fig 2 (The map was created using open-access data from Natural Earth (naturalearthdata.com)).

### 2.2 Research methodology

#### 2.2.1 Global frozen meat network construction.

The global frozen meat trade countries (regions) are treated as network nodes, with trade relations between countries (regions) forming the edges. The trade volume and trade intensity distance serve as the edge weights, constructing a complex network model. The global frozen meat trade network is defined as follows:


$$\begin{array}{c} \begin{array}{c} G_{m}=\left(V,E,W^{\text{1}},W^{\text{2}}\right) \end{array} \end{array}$$ (1)

Where: m represents the four different global frozen meat networks: beef, pork, mutton, and poultry. V is the set of nodes, representing all countries (regions). E is the set of edges, representing trade relations between countries (regions). $W^{\text{1}}$ is the set of edge weights based on trade volume between all nodes. $W^{\text{2}}$ is the set of edge weights based on trade intensity distance between all nodes.

Trade volume (in kilograms) is selected as the edge weight for modeling, in order to avoid the effects of inflation and price fluctuations, thus providing a true representation of supply chain trade relationships and dependencies, which is crucial for analyzing network characteristics. At the same time, following existing research [62], trade intensity distance is introduced as an edge weight to explore its impact on the shortest path length. The specific formula for this edge weight is as follows:


$$\begin{array}{c} w_{ij}^{\text{2}}=\text{1}+lnMax\left(w_{ij}^{\text{1}}\right)-ln\left(w_{ij}^{\text{1}}\right) \end{array}$$ (2)

Where: $w_{ij}^{\text{2}}$ represents the trade intensity distance between node i and node j. $Max\left(w_{ij}^{\text{1}}\right)$ represents the maximum trade volume between any two nodes in the network. $w_{ij}^{\text{1}}$ represents the trade volume of frozen meat exported from node i and node j.

In real-world scenarios, trade intensity distance is undoubtedly influenced by more complex factors, such as political relations, geographical location, cultural differences, and many other hard to quantify elements between two countries. However, within the specific analytical framework of this paper, we focus solely on trade volume as the core factor.

#### 2.2.2 Weighted Network Characteristics Indicators Involved.

Before conducting the network resilience analysis, we first perform an analysis of the topology and trade group evolution of the frozen meat directed weighted trade network. The relevant indicators are shown in Table 3 below. All the indicators in this table are weighted metrics.

**Table 3:** Indicators of complex network related features.

Indicator	Formulae	Meaning
Network density	$\begin{array}{c} \rho =\frac{E}{V(V-\text{1})} \end{array}$ (3)	Network density, ρ, is the ratio of the actual number of directed edges E in the graph to the maximum possible number of directed edges between all pairs of nodes *V(V− 1)*.
Node out-strength	$\begin{array}{c} W_{i}^{out}=\sum_{j=\text{1}}^{N}\;w_{ij} \end{array}$ (4)	Node out-strength [63], $W_{i}^{\;out}$, refers to the sum of the trade volume weights ($w_{ij}$) that node *w*_*ij*_ exports to all other nodes.
Node in-strength	$\begin{array}{c} W_{i}^{in}=\sum_{j=\text{1}}^{N}\;w_{ji} \end{array}$ (5)	Node in-strength, $W_{i}^{\;in}$, refers to the sum of the trade volume weights ($w_{ji}$) that node *w*_*ij*_ imports from all other nodes.
Betweenness centrality	$\begin{array}{c} BC_{w^{2}\;i}=\sum_{s\neq i\neq t}\;\frac{n_{w^{2}\;st}^{i}}{g_{w^{2}\;st}} \end{array}$ (6)	Weighted betweenness centrality [64], $BC_{w_{i}^{\text{2}}}$, measures the importance and influence of a country (region) in the network, considering the trade intensity distance weight. It is the number of weighted shortest paths passing through node i, $n_{w^{\text{2}}st}^{i}\;$divided by the total number of weighted shortest paths between other trade pairs, $g_{w^{\text{2}}st}$. This metric captures the “bridge” role of a node in the network.
Closeness centrality	$\begin{array}{c} CC_{w^{\text{2}}i}=\frac{\text{1}}{d_{i}}=\frac{N}{\sum_{j=\text{1}}^{N}\;d_{w_{ij}^{\text{2}}}} \end{array}$ (7)	Weighted closeness centrality [63], $CC_{w_{i}^{\text{2}}}$, is the inverse of the average weighted shortest path distance $d_{w_{ij}^{\text{2}}}$ from node i to all other nodes, considering trade intensity distance weights. It measures the importance of a node in the network based on its average distance to all other nodes.
Modularity	$\begin{array}{c} Q=\frac{\text{1}}{\text{2}H}\;\sum_{ij}[\;w_{ij}^{\text{1}}\;-\;\frac{\;k_{w_{i}}k_{w_{j}^{\text{1}}}}{\text{2}H}\;,\delta \left(c_{i},c_{j}\right) \end{array}$ (8)	Modularity, Q, measures the difference in connection strength between nodes within a community and the expected connection strength between nodes in a random network, considering trade volume weights. Specifically, the modularity formula calculates the difference between the connection weight $w_{ij}^{\text{1}}$ of node pairs i and j. in the network and their expected connection weight under random conditions,$\frac{\;k_{w_{i}}k_{w_{j}^{\text{1}}}}{\text{2}H}$, and perform the summation only for node pairs within the same community. The function $\delta \left(c_{i},c_{j}\right)$ ensures that only contributions from node pairs within the same community are counted. Here, $c_{i}$ and $c_{j}$ are the communities of node i and j, and δ=1 if $c_{i}=c_{j}$, otherwise δ=0.

#### 2.2.3. Static structural resilience indicators.

Structural resilience refers to the ability of a network to maintain its overall functionality and continue to operate despite encountering failures, attacks, or disruptions. This article comprehensively analyzes the structural resilience of the global frozen meat trade network from four dimensions: transmissibility, clustering, hierarchy, and assortativity. Four indicators—global efficiency, average clustering coefficient, degree distribution, and assortativity coefficient—are selected to perform resilience analysis in both unweighted and weighted forms for each indicator. In the unweighted network, we focus on the existence of trade connections between countries, outlining the structure of the frozen meat trade network. The weighted network introduces trade volume or trade strength distance as weights to more precisely measure interaction intensity, revealing deeper information and patterns. The specific indicators are shown in Table 4.

**Table 4:** Static Structural Resilience Indicators.

Indicator		Formulae
Transmissibility	global efficiency	$\begin{array}{c} E=\frac{\text{1}}{N(N-\text{1})}\sum_{i\neq j}\;\frac{\text{1}}{d_{ij}} \end{array}$ (9)
		$\begin{array}{c} E_{w^{2}}=\frac{\text{1}}{N(N-\text{1})}\sum_{i\neq j}\;\frac{\text{1}}{d_{w_{ij}^{\text{2}}}} \end{array}$ (10)
	Global efficiency [65], E, is an indicator that measures the efficiency of information transmission within a network. It is typically defined as the average of the inverse sum of the shortest path lengths $d_{ij}$ between all pairs of nodes in the network. Weighted global efficiency, *E*_*w*^2^_, calculates the average of the inverse sum of the shortest path lengths weighted by trade intensity distance $d_{w_{ij}^{\text{2}}}$. N is the total number of nodes in the network.	
Clustering	average clustering coefficient	$\begin{array}{c} C_{i}=\frac{\text{1}}{k_{i}\left(k_{i}-\text{1}\right)}\sum_{s\neq t}^{s,t\;are\;adjacent\;to\;i}d_{st} \end{array}$ (11) $\begin{array}{c} C=\frac{\text{1}}{N}\sum_{i=\text{1}}^{N}\;C_{i} \end{array}$ (12)
		$\begin{array}{c} C_{w_{i}}=\frac{\text{1}}{k_{i}\left(k_{i}-\text{1}\right)Max\left(W_{ij}\right)}\sum_{s\neq t}^{s,tare\;adjacent\;to\;i}d_{wst} \end{array}$ (13) $\begin{array}{c} C_{w}=\frac{\text{1}}{N}\sum_{i=\text{1}}^{N}\;C_{w_{i}} \end{array}$ (14)
	Weighted average clustering coefficient [66], *C*_*w*_, calculates the total sum of the actual trade volumes $d_{wst}$ between a node i ‘s neighboring nodes s and t, divided by the maximum possible sum of trade volumes that could exist between the neighbors, $k_{i}\left(k_{i}-\text{1}\right)Max\left(W_{ij}\right)$, and then takes the arithmetic mean of the weighted clustering coefficients $C_{w_{i}}$ for all nodes. The unweighted clustering coefficient, C, does not consider the influence of weights.	
Hierarchy	degree distribution	$\begin{array}{c} k_{i}=c{\left(k_{i}^{*}\right)}^{\alpha } \end{array}$ (15) $\begin{array}{c} lnk_{i}=lnc+\alpha (k_{i}^{*}) \end{array}$ (16)
		$\begin{array}{c} k_{w_{i}}=c_{w^{\text{1}}}{\left(k_{w_{i}}^{*}\right)}^{\alpha } \end{array}$ (17) $\begin{array}{c} {lnk}_{w_{i}}=lnc_{w^{\text{1}}}+\alpha \left(k_{w_{i}^{1}}^{*}\right) \end{array}$ (18)
	Here,$k_{i}$ is the degree of node i; $k_{w_{i}}$ is the trade volume weighted degree of node i; $k_{i}^{*}$ is the ranking of node i’s degree in the network; $k_{w_{i}}^{*}$is the ranking of node i’s trade volume weighted degree in the network; c and $c_{w^{\text{1}}}$ is a constant; and α is the slope of the degree distribution curve [66].	
Assortativity	Assortativity coefficient	$\begin{array}{c} AC=\frac{M^{-\text{1}}\sum_{i}\;k_{s}k_{t}-(M^{-\text{1}}\sum_{i}\;\frac{\text{1}}{\text{2}}k_{s}+k_{t}{)}^{\text{2}}}{M^{-\text{1}}\sum_{i}\;\frac{\text{1}}{\text{2}}\left(k_{s}^{\text{2}}+k_{t}^{\text{2}}\right)-{\left[M^{-\text{1}}\sum_{i}\;\frac{\text{1}}{\text{2}}\left(k_{s}+k_{t}\right)\right]}^{\text{2}}} \end{array}$ (19)
		$\begin{array}{c} AC_{w^{1}}=\frac{H^{-\text{1}}\sum_{i}\;w_{i}\left(k_{s}k_{t}\right)-{\left[H^{-\text{1}}\sum_{i}\;\frac{\text{1}}{\text{2}}w_{i}\left(k_{s}+k_{t}\right)\right]}^{\text{2}}}{H^{-\text{1}}\sum_{i}\;\frac{\text{1}}{\text{2}}w_{i}\left(k_{s}^{\text{2}}+k_{t}^{\text{2}}\right)-{\left[H^{-\text{1}}\sum_{i}\;\frac{\text{1}}{\text{2}}w_{i}\left(k_{s}+k_{t}\right)\right]}^{\text{2}}} \end{array}$ (20)
	AC is the assortativity coefficient, *AC*_*w*^1^_is the trade-weighted assortativity coefficient;M is the total number of edges; $H=\sum_{i}\;w_{i}$ is the sum of all edge trade volume weighted values; $k_{s}$ and $k_{t}$ are the degrees of nodes s and t, respectively; and $w_{i}\;$represents the trade weight of the i-th edge [67].	

#### 2.2.4 Dynamic structural resilience indicators.

Dynamic structural resilience of networks is a comprehensive concept that involves the vulnerability and invulnerability exhibited by the network when faced with challenges. Vulnerability refers to the potential for information disruption, resource imbalance, or even network paralysis due to dependence on key nodes, links, or insufficient redundancy in path design when the network encounters internal or external disturbances. In contrast, invulnerability refers to the network’s ability to maintain its performance or functionality under impact. This paper evaluates and compares the dynamic structural resilience of unweighted and weighted directed networks from the two dimensions of vulnerability and invulnerability. The relevant indicators are shown in Table 5.

**Table 5:** Dynamic structural resilience indicators.

Indicator		Formulae
Vulnerability	loss rate of network performance caused by single node interruption	$\begin{array}{c} {L_{E}}_{i}=\left(\text{1}-\frac{E_{i}}{E}\right)\times \text{1}\text{0}\text{0}\% \end{array}$ (21) $\begin{array}{c} L_{E_{w}2_{i}}=\left(\text{1}-\frac{E_{w^{2}i}}{E_{w^{2}}}\right)\times \text{1}\text{0}\text{0}\% \end{array}$ (22)
		$\begin{array}{c} L_{D_{i}}=\left(\text{1}-\frac{D_{i}}{D}\right)\times \text{1}\text{0}\text{0}\% \end{array}$ (23) $\begin{array}{c} L_{D_{w^{1}i}}=\left(\text{1}-\frac{D_{w^{1}i}}{D_{w^{1}}}\right)\times \text{1}\text{0}\text{0}\% \end{array}$ (24)
	The network loss rate based on unweighted global efficiency *L*_*E*_*i*__ is equal to 1 minus the quotient of the unweighted global efficiency of the network $E_{i}$ after node i is disrupted and the initial global efficiency of the network E. The network loss rate based on weighted global efficiency $L_{E_{w^{2}i}}$ is equal to 1 minus the quotient of the unweighted global efficiency of the network $E_{w^{2}i}$ after node i is disrupted and the initial global efficiency of the network $E_{w^{2}i}$. The network loss rate based on node degree *L*_*D*_*i*__ is equal to 1 minus the quotient of the unweighted node degree of the network $D_{i}$ after node i is disrupted and the initial node degree D. The network loss rate based on total strength $L_{D_{w^{1}i}}$ is equal to 1 minus the quotient of the total strength $D_{w^{1}i}$ after node i is disrupted and the initial total strength *D*_*w*^1^_.	
Invulnerability	average retention rate of network performance	$\begin{array}{c} P_{E}=\frac{\sum_{i=1}^{n}\;P_{E_{i}}}{n}\times \text{1}\text{0}\text{0}\% \end{array}$ (25) $\begin{array}{c} P_{E_{w^{2}}}=\frac{\sum_{i=1}^{n}\;P_{E_{w^{2}i}}}{n}\times \text{1}\text{0}\text{0}\% \end{array}$ (26)
		$\begin{array}{c} P_{D}=\frac{\sum_{i=1}^{n}\;P_{D_{i}}}{n}\times \text{1}\text{0}\text{0}\% \end{array}$ (27) $\begin{array}{c} P_{D_{w^{i}}}=\frac{\sum_{i=1}^{n}\;P_{D_{w^{1}i}}}{n}\times \text{1}\text{0}\text{0}\% \end{array}$ (28)
	The average retention rate of global efficiency $P_{E}$ in the network refers to the mean value of the global efficiency retention rate *P*_*E*_*i*__ after the interruption of each of the n core nodes in the network. The average retention rate of weighted global efficiency *P*_*E*_*w*^2^__ refers to the mean value of the weighted global efficiency retention rate $P_{E_{w^{2}i}}$ after the interruption of each of the n core nodes. These two indicators measure the average retention level of the overall network connectivity. Similarly, the average retention rate of node degree $P_{D}$ and the average retention rate of total strength *P*_*D*_*w*^1^__ refer to the average connection scale retention rates *P*_*D*_*i*__ and $P_{D_{w^{1}i}}$ after the interruption of each of the n core nodes in the network, respectively. In this study, n=10.	

### 2.3. Data sources and processing

This study utilizes data from the United Nations Commodity Trade Statistics Database The dataset necessary to replicate the study’s findings has been deposited in the recommended stable public repository Figshare. Spanning from 2003 to 2023, covering over 170,000 trade records between 232 countries (or regions) worldwide. For consistency in the data, the study selects import data and addresses issues such as missing data and regional consolidation (combining data from Hong Kong and Macau into China, but excluding Taiwan). Finally, the trade data is categorized and aggregated by the four major types of meat: beef, pork, mutton, and poultry.

## 3. Result analysis

### 3.1. Evolution of the pattern of the global frozen meat trade network

#### 3.1.1. Descriptive statistical analysis of the trade network.

**Fig 3:**
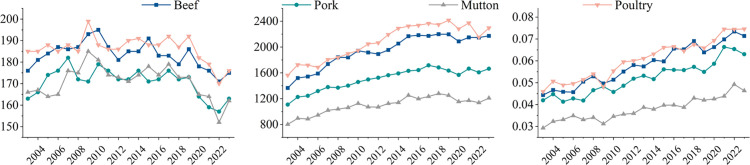
Time series graph of the number of nodes, number of edges, and network density in the trade network from 2003 - 2023. **(a)** Nodes. **(b)** Edges. **(c)** Network density.

Fig 3 presents the statistical characteristics of the global frozen meat trade network from 2003 to 2023. The number of trade nodes exhibits a downward trend during this period, with a more pronounced decline following the outbreak of the global COVID-19 pandemic in 2020. However, the number of trade edges and network density continue to show an upward trend, reflecting the deepening trade ties between core countries and the optimization of the trade network. This also highlights the challenges that globalization may pose to smaller or peripheral countries.

**Fig 4:**
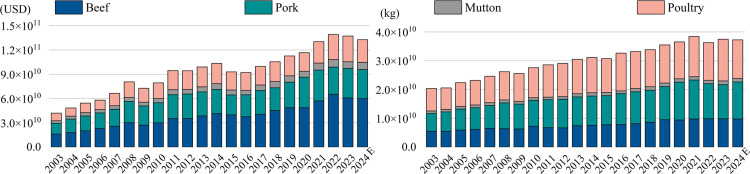
Temporal variation in the value and volume from 2003 - 2024. (a) Trade value. (b) Trade volume.

Fig 4 depicts the overall changing trends of the global trade volume and trade value of frozen meat from 2003 to 2024, among which the data for 2024 is estimates.

As shown in Fig 4, during the study period, global frozen meat trade exhibited a significant expansion in scale, with both trade volume and trade value achieving dual growth amid fluctuations. Specifically, the total value of global frozen meat trade increased from $41.959 billion at the base period to $137.207 billion, with an average annual compound growth rate of 11.4%. The trade volume grew from 20.349 billion kilograms to 37.468 billion kilograms, with an average annual growth rate of 7.0%. It is noteworthy that, with a relatively low unit price, the trade value of poultry meat only accounts for 22.8% of the total trade value, while its trade volume accounts for more than 37.8%. In contrast, beef trade displayed a reverse pattern, with its high added-value attribute driving its trade value share to exceed 40%, while its trade volume share was only about 25%. Given that price fluctuations and inflation factors could potentially interfere with trade value data, this study chooses trade volume as the core analytical indicator to systematically reveal the dynamic evolution of the global frozen meat trade network’s topology and resilience.

#### 3.1.2. Evolutionary analysis of trade network topology.

To clearly illustrate the topological characteristics of the global frozen meat trade network, this study utilizes flow chord diagrams to visualize the trade link features of four categories—pork, beef, mutton, and poultry—for the years 2003 and 2023. In these diagrams, the thickness of the arcs represents the trade volume of each country (or region), while the arrows indicate the direction of trade flow.

**Fig 5:**
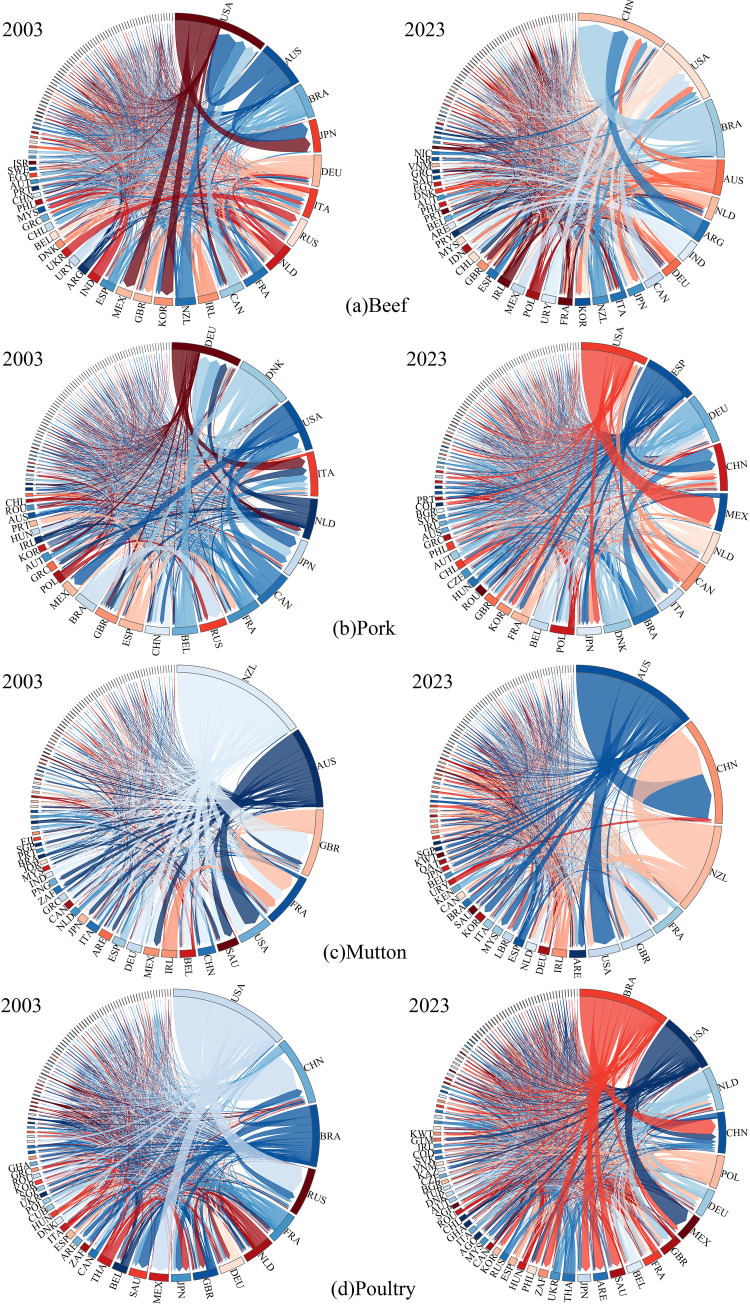
The topological structure of the trade network in 2003 and 2023.

As shown in Fig 5, the beef trade pattern experienced significant changes. In 2003, Australia and the United States dominated the market, but by 2023, exports from Brazil to China had become the largest trade flow, with China emerging as the largest importer and the United States remaining an important node. In pork trade, the early period was characterized by imports from Italy and Germany and exports mainly from Denmark and the United States. By 2023, the trade pattern shifted to China being the dominant importer and Spain the leading exporter, with the United States exporting large quantities to Mexico. For mutton exports, New Zealand and Australia redirected their trade from traditional European markets to China, with China and the United States becoming the main importers. In poultry trade, the United States was the largest exporter to China in 2003, but by 2023, Brazil surpassed the United States as the leading exporter, with its primary exports directed to China.

**Fig 6:**
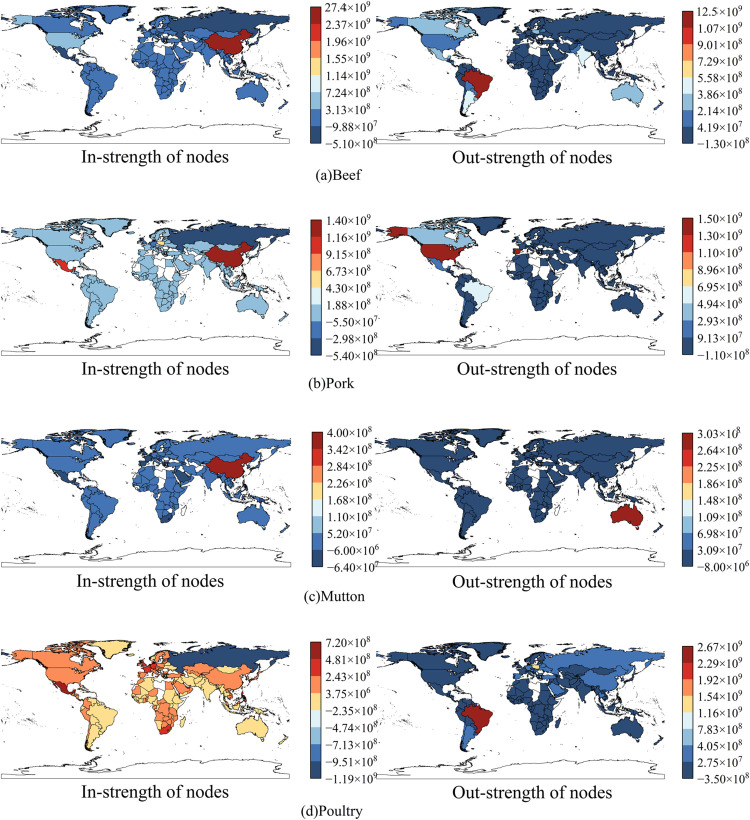
Grading map of the change values of the node strength in trade network in 2003 and 2023.

To clearly demonstrate the evolution of frozen meat node strength across countries, this study compares trade volume changes between 2003 and 2023, classifies the changes according to magnitude, and presents the results through a graded map, as shown in Fig 6 (The map was created using open-access data from Natural Earth (naturalearthdata.com)).

Overall, as shown in Fig 6, the number of nodes with increased trade strength is greater than those with decreased trade strength, indicating a noticeable expansion trend in the trade network. In Fig 6(a), traditional beef exporters such as Brazil and Argentina show significant changes in export strength, maintaining their important positions in global beef trade. The increased demand for beef in the United States and Germany results in a rise in import strength. In Fig 6(b), China has increased pork imports while reducing exports, whereas Germany shows the opposite trend, with Spain significantly enhancing its export strength. In Fig 6(b), in the lamb trade, China serves as the core node of import fluctuations, while Australia occupies a dominant position on the export side, highlighting its significance as a major supplier. In Fig 6(d), both the United States and Brazil experience substantial changes in poultry export stren gth, reflecting their competitive advantages in the global poultry market.

Additionally, there are notable differences in performance across countries in different meat trade categories. China, as a core importing country, plays an important role in the trade of beef, pork, and lamb. On the supply side, Brazil (beef, poultry), the United States (poultry), and Australia (lamb) occupy a dominant position in different categories respectively, reflecting the significant characteristics of the supply and demand pattern and national differences in the global frozen meat trade.

**Fig 7:**
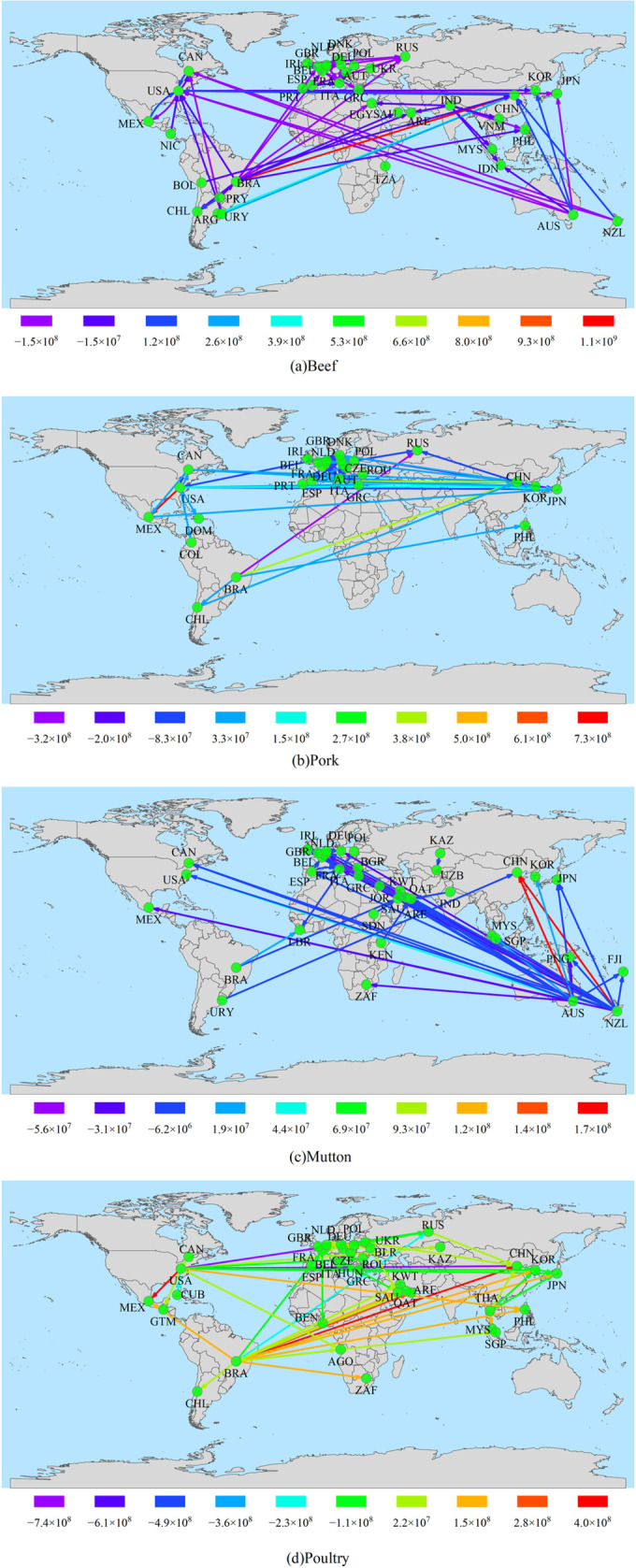
Map of flow changes of main trade nodes and their connected edges in trade network in 2003 and 2023.

In order to intuitively present the changes in the trade volume of the main bilateral trade within the global frozen meat trade network in 2003 and 2023, this article has drawn a map of trade flow changes, and distinguished the flow change amounts of each connection through different colors. The results are shown in Fig 7 (The map was created using open-access data from Natural Earth (naturalearthdata.com)).

As shown in Fig 7, China has become the core node for beef imports, with exports from Brazil surging by 1.19 billion kilograms and exports from Argentina increasing by 523 million kilograms. In contrast, traditional exporters such as Australia and New Zealand have significantly reduced their trade with the United States.

In the pork network, China’s dependence on Spain and Brazil has risen rapidly. Exports from the United States to Mexico have increased by 841 million kilograms, making it the largest trade flow. Exports from Brazil and traditional European exporters to Russia have significantly decreased. Germany has expanded its market to Eastern Europe, with Spain emerging as a new export hub in Europe, experiencing a surge in trade volume with the Asia-Pacific market.

In the mutton trade, New Zealand’s exports to Europe have generally decreased, but exports to emerging markets in Asia and the Middle East have grown significantly. For example, exports to the United Kingdom decreased by 55.29 million kilograms, while exports to China increased by 190 million kilograms. Australia, on the other hand, has expanded its trade in North America (the United States) and East Asia (China, South Korea).

For poultry meat, China has increased its imports from Brazil. The United States saw a rise of 444 million kilograms in imports from Mexico, while its exports to China and Russia declined by 713 million kilograms and 735 million kilograms respectively. Meanwhile, Brazil has emerged as a core export node, with a substantial surge in its trade volume to the United Arab Emirates (UAE), Japan, Mexico, and South Korea.

#### 3.1.3 Evolutionary analysis of trade network node centrality.

**Fig 8:**
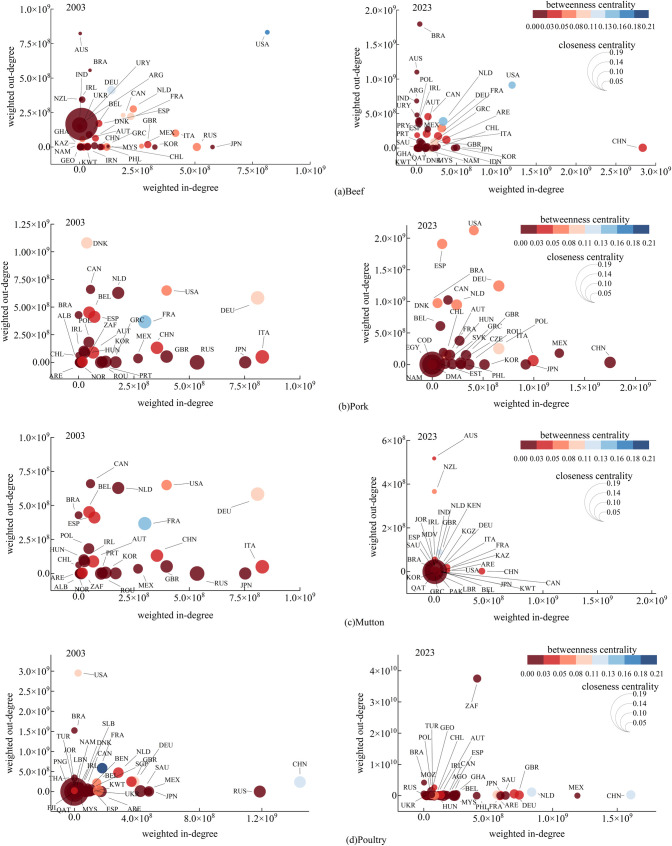
Evolution of node centrality in the weighted trade network in 2003 and 2023.

To gain a deeper understanding of the evolution of the frozen meat trade network, particularly the changes in the status and influence of core nodes, we further conducted a comparative analysis of the centrality of key nodes within the frozen meat trade network. The results are shown in Fig 8.

A comparison of data from 2003 to 2023 reveals that the global frozen meat trade network has become “multi-polar,” with regional hubs rising to reflect a parallel process of globalization and regionalization. Specifically:

Beef: The United States has moved up significantly in terms of weighted indegree and intermediary centrality, ranking from 14th to 2nd, showing a notable improvement in its status. China has transitioned from a peripheral importer to the world’s largest import hub, with its intermediary centrality rising. The Netherlands has strengthened its transshipment function, with both intermediary centrality and overall ranking rising to 1st, making it the most influential. Germany, France, and the United Kingdom have maintained high rankings in weighted indegree and intermediary centrality, with their comprehensive ranking within the top 10. Brazil’s strong export capacity has pushed its weighted outdegree to 1st place. The United Arab Emirates (UAE) secured the top position in the closeness centrality ranking in 2023, highlighting its role as a regional hub.

Pork: China has seen rapid growth in both its weighted indegree and intermediary capacity. The United States continues to maintain dual advantages in exports and intermediary roles. Germany’s weighted indegree has slightly decreased but remains among the top, with a stable overall position. Paraguay’s rising closeness centrality has made it a key node in the network. Denmark’s weighted outdegree and intermediary centrality have slightly declined but remain high. Spain has expanded its exports, become the second-largest exporter only after the United States.

Mutton: The United Arab Emirates ranked first in the 2023 comprehensive ranking, with improvements across all metrics, highlighting its significance. China has jumped to the top in terms of weighted indegree, with a significant increase in intermediary centrality, becoming an absolute hub. The United States has seen growth in weighted indegree, but its influence on trade routes has weakened. The United Arab Emirates has emerged as a new trading center, with active imports and an intermediary role. Uzbekistan’s closeness centrality rises to the top, with high efficiency in information transmission. Australia has surpassed New Zealand to become the largest exporter.

Poultry: China ranked first in 2003, with multiple indicators in the top 3, and remains a core player, ranking second in 2023. The United States has seen growth in weighted outdegree, though its intermediary centrality has decreased. The Netherlands performed exceptionally in 2023, ranking first overall and betweenness centrality metrics, with its weighted indegree and outdegree ranking 4th and 5th respectively, emerging as a new core player. The United Arab Emirates has strengthened its control and become a new hub, while traditional strongholds like France have seen a decline in their status. Brazil has strengthened its export capacity, although its betweenness centrality has not risen in tandem. Germany’s export capacity has steadily grown, with betweenness centrality ranking 7th, maintaining its position as a European hub. Changes in the network structure have caused Fiji’s centrality to drop significantly, with its hub status has been replaced by the Democratic Republic of the Congo.

#### 3.1.4 Evolutionary analysis of trade network community division.

**Fig 9:**
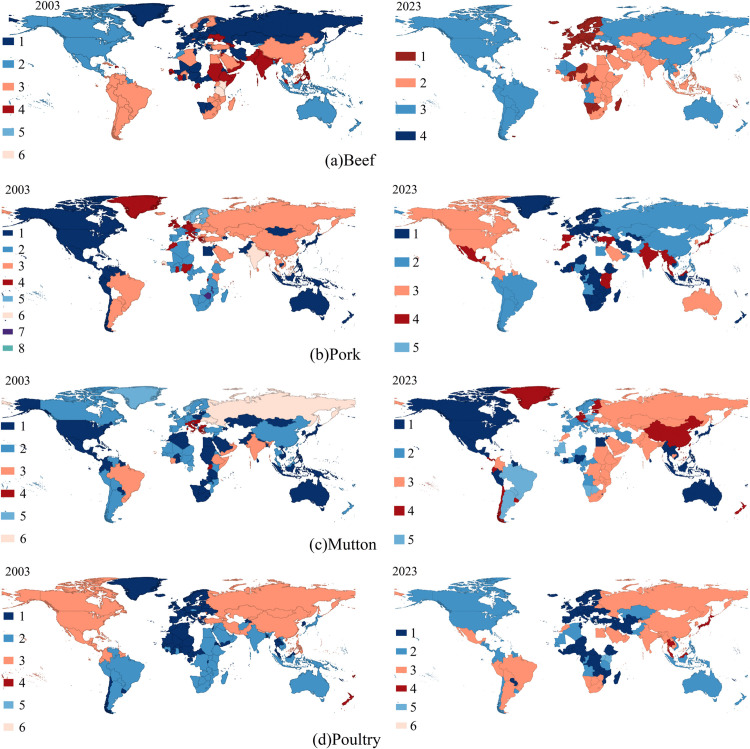
Geographic distribution of trade communities in 2003 and 2023. (The map was created using open-access data from Natural Earth (naturalearthdata.com))illustrates the evolution of the global frozen meat trade network’s community structure from 2003 to 2023, with the following details.

To further reveal the clustering relationships and community structure characteristics among countries (regions) within the network, this study applies the Louvain community detection algorithm to divide the trade communities of 2003 and 2023, with the aim of comparing and analyzing the evolutionary trends of these trade communities over the two years [68], The results are shown in Fig 9.

During this period, the number of communities in the beef trade network remained unchanged. The largest community, primarily composed of European countries, had 52 and 59 member countries in 2003 and 2023, respectively. Russia, Mongolia, and Kazakhstan separated from this community and joined other communities. The communities consisting of North America and Oceania, as well as those led by China and Brazil, both saw a reduction in size.

The pork trade network became more regionally concentrated, with the number of communities decreasing from 8 to 5. Several smaller communities merged into larger ones, such as Peru and Chile joining the community led by China, Russia, and Brazil. Australia separated from Community 1 and formed another community together with Western European, Central African countries and others, boasting 32 member states.

In the mutton trade network, Community 6, dominated by Russia and other countries, was merged into Community 3. Canada and Australia separated from Community 1 and joined Community 2.

The poultry trade network was composed of three major communities and several smaller ones. The number of countries (regions) in community 1 decreased from 77 to 67. Community 3, dominated by Asian and North American countries, split into two communities: one consisting of North America and Oceania, and the other consisting of South America and East Asia.

### 3.2 Evolution of static structural resilience in the global frozen meat trade network

#### 3.2.1 Transmissibility evolution analysis.

**Fig 10:**
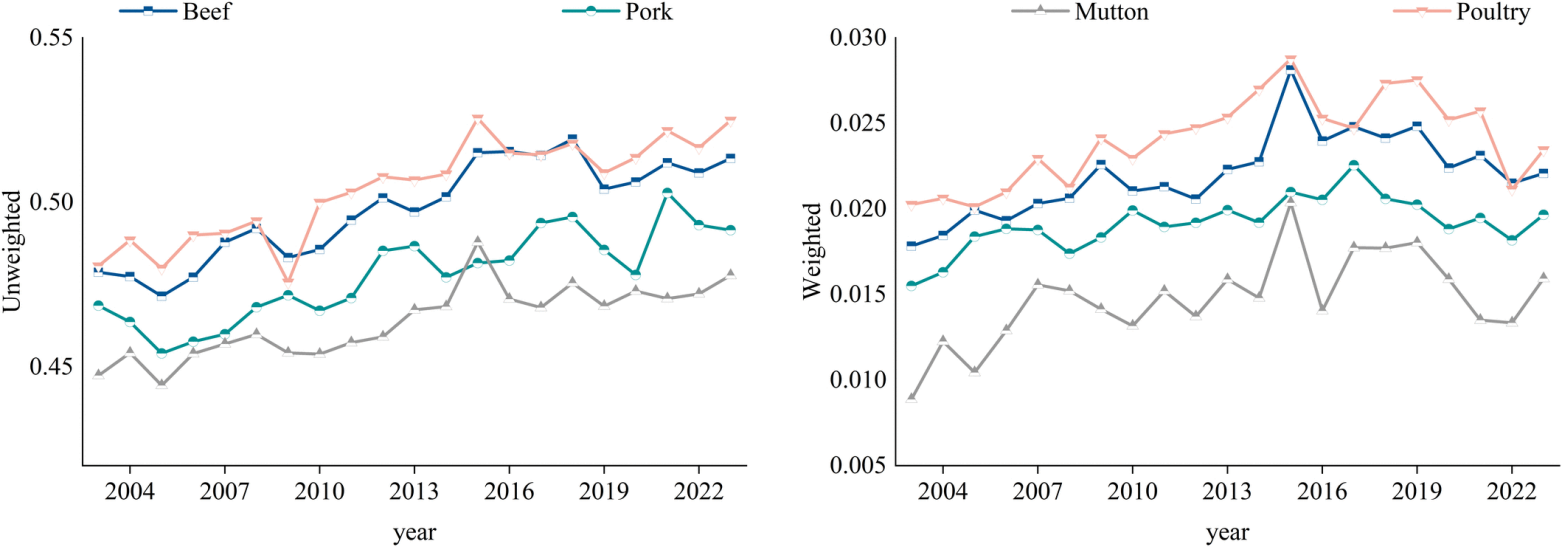
Evolution of the global efficiency of the trade network. **(a)** Unweighted. **(b)** Weighted.

Fig 10 shows the evolution of global efficiency in the global frozen meat trade network from 2003 to 2023. From the Fig 10, it can be seen that the trends of both weighted and unweighted global efficiency in the four frozen meat trade networks are largely consistent, both showing an upward trend. This indicates that the transmissibility of the network has been continuously improving, and the connectivity and circulation of frozen meat trade have been significantly enhanced. In general, the global efficiency of poultry, beef, pork, and mutton trade decreases in this order, which aligns with the network density ranking, suggesting that there is a positive correlation between network transmissibility and network density.

#### 3.2.2 Clustering evolution analysis.

**Fig 11:**
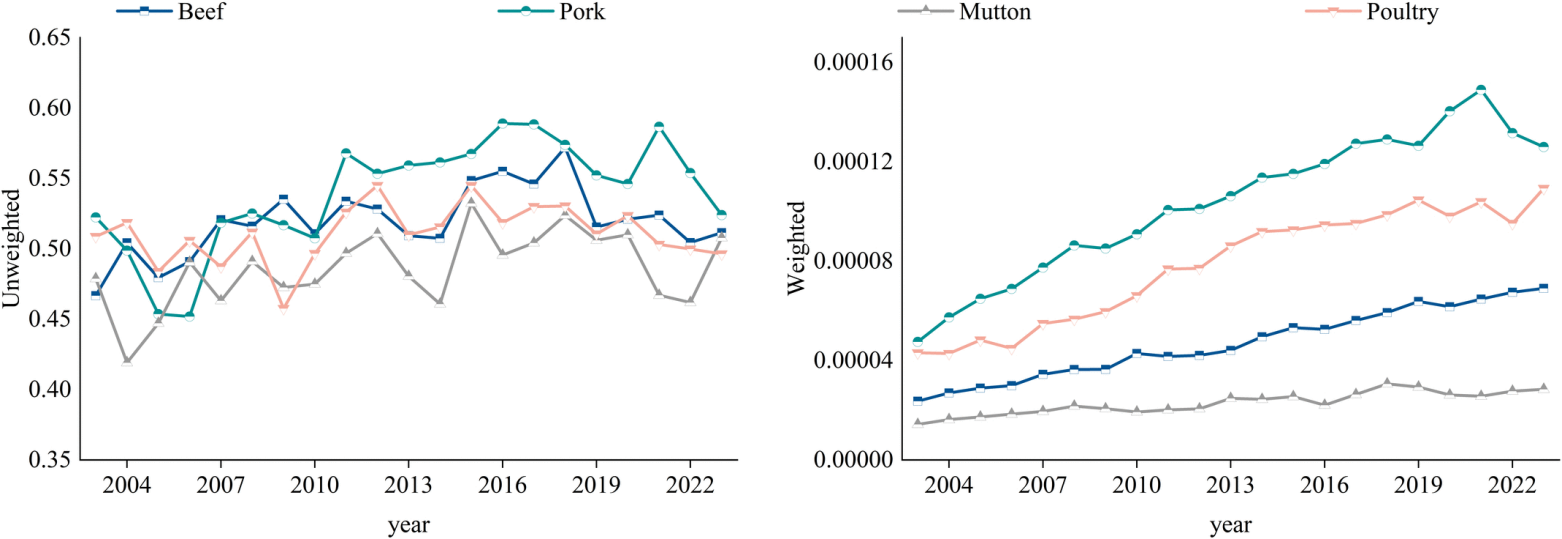
Evolution of the average clustering coefficient of the trade network. **(a)** Unweighted. **(b)** Weighted.

Fig 11 illustrates the evolution of the average clustering coefficient in the global frozen meat trade network from 2003 to 2023. The trends of both weighted and unweighted average clustering coefficients are mostly the same. Notably, pork and poultry show a clear upward trend, while beef and mutton exhibit a more gradual increase, indicating that the trade networks of pork and poultry have formed tighter clustering structures over the study period. In contrast, beef and mutton trade partners have remained relatively stable, with little change. Furthermore, the unweighted average clustering coefficient fluctuates significantly, while the weighted average clustering coefficient rises steadily with minimal fluctuation. There is also a clear stratification in the weighted average clustering coefficients across different meat types. This suggests that the frozen meat trade network is continuously adjusting dynamically. The fluctuations in clustering coefficients are driven by the dynamic changes in peripheral and new nodes, but the trade volume among core nodes continues to grow steadily, forming an inertia effect of trade volume weight.

#### 3.2.3 Hierarchy evolution analysis.

**Fig 12:**
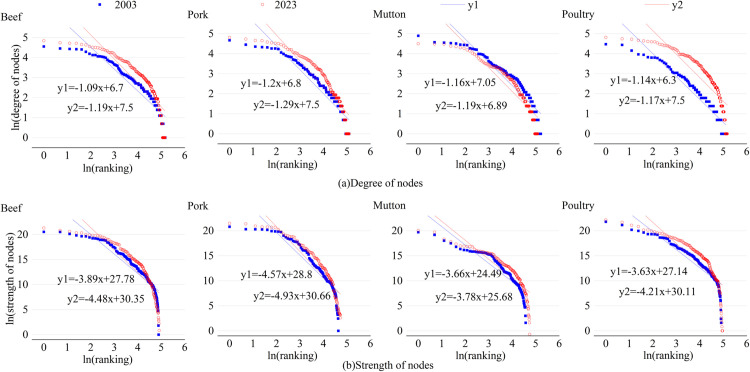
Evolution of the degree distribution of the trade network.

This paper provides an in-depth analysis of the degree distribution characteristics of the four frozen meat trade networks, both weighted and unweighted, to visually present the network’s hierarchical structure. The results are shown in Fig 12.

The degree distributions of the four frozen meat trade networks all follow a power-law distribution, reflecting the characteristics of scale-free networks. Among them, the degree distribution of the weighted networks is display a steeper hierarchy than that of the unweighted networks. This indicates that a few nodes control a disproportionately high trade volume, while the majority of nodes have relatively lower trade volumes. Comparing data across different years, the power-law exponents of both unweighted and weighted networks have increased. Specifically, in 2023, the power-law exponents for the weighted degree distributions of pork and beef networks were 4.93 and 4.48, respectively, highlighting a strong hierarchical structure. The power-law exponents for mutton and poultry networks were 3.78 and 4.21, indicating a more flattened network structure.

#### 3.2.4 Assortativity evolution analysis.

**Fig 13:**
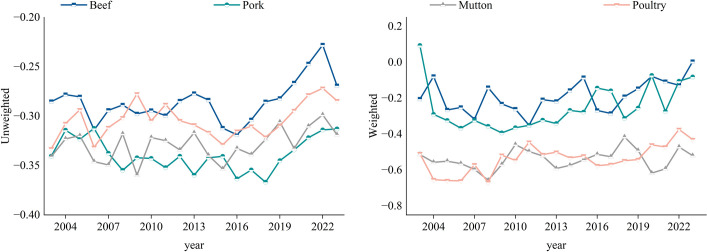
Evolution of the assortativity coefficient of the trade network. **(a)** Unweighted. **(b)** Weighted.

The assortativity of a network is a key metric for measuring whether nodes tend to connect with similar nodes, revealing the connection preferences in trade networks. In this study, the assortativity coefficient is used for measurement. When the assortativity coefficient is positive, high-degree (or high-trade volume) nodes are more likely to connect with each other, forming a tightly-knit core group. When the coefficient is negative, low-degree (or low-trade volume) nodes connect to high-degree nodes through hubs, resulting in a more decentralized network structure. The results are shown in Fig 13.

Only the weighted assortativity coefficients of pork in 2003 and beef in 2023 briefly exceeded 0, while all other assortativity coefficients were negative. This reveals the disassortative nature of these networks. Observing the data in Fig 13(a), it is clear that the assortativity coefficients for beef and pork fluctuate within the range of 0 to −0.3, while for mutton and poultry, the coefficients fluctuate between −0.3 and −0.6. This suggests that the global frozen meat trade network is not a static structure, but rather one that is continually evolving and adjusting. The dependence of “small nodes” on “hubs” also changes as various factors evolve.

### 3.3 Simulation analysis of dynamic structural resilience in global frozen meat trade network

To explore the evolutionary characteristics of dynamic structural resilience in trade networks, this study employs a dual-dimensional comparative framework of unweighted and weighted networks, examining the following dimensions: (1) quantifying the network performance loss rate caused by the failure of a single node based on 2023 cross-sectional data; (2) identifying vulnerable nodes through temporal evolution data analysis, revealing the evolutionary patterns of the maximum performance loss rate resulting from node removal; (3) assessing the mean performance loss in network performance under the failure scenario of core nodes, and analyzing the role of key nodes in system stability. The study focuses on examining the two key indicator systems: global efficiency loss rate and node degree/strength loss rate.

#### 3.3.1 Simulation analysis of single node interruption network performance loss rate.

**Fig 14:**
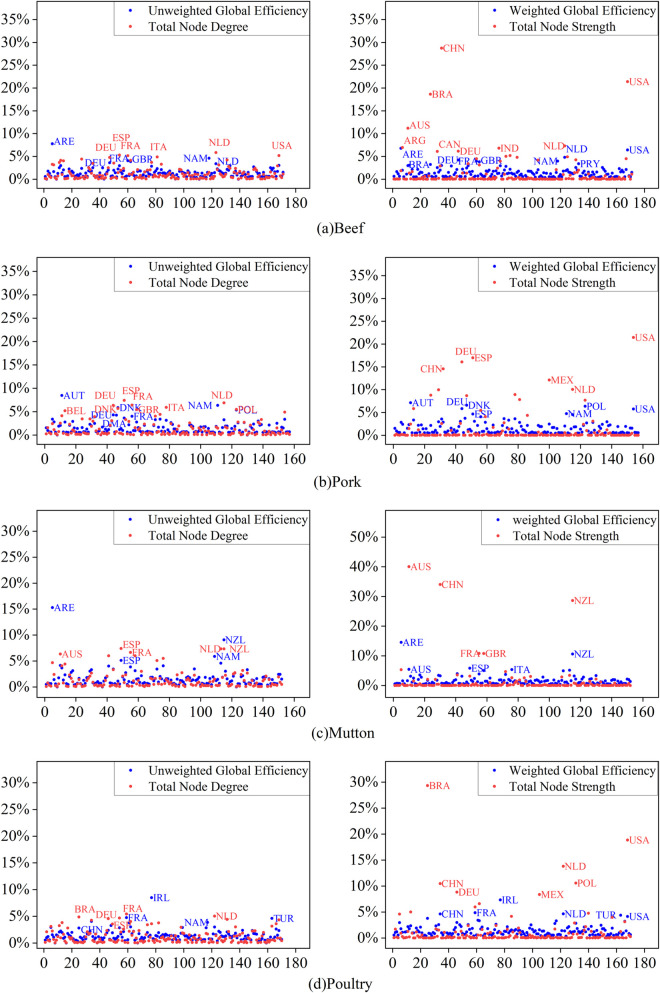
Performance loss rate of the trade network based on single node disruption in 2023.

Through disruption simulation, the global efficiency loss rate and node strength loss rate at the single node failure of each node in the frozen meat trade networks of beef, pork, mutton, and poultry in 2023 were sequentially calculated. The results are shown in Fig 14. Similar to the previous studies, this research also analyzes both the unweighted and weighted network dimensions.

Visual analysis of simulation data revealed that in the unweighted trade networks for beef, pork, mutton, and poultry, nodes whose failure resulted in a network performance loss rate greater than 5% accounted for 0.5%, 2.4%, 2.4%, and 0.5% of their respective networks. For the same loss rate range in the weighted trade networks of the four meat types, the proportions of failed nodes were 1.1%, 3.0%, 4.9%, and 0.5%, respectively. These figures reflect the scenario for global efficiency loss rates. In contrast, the proportion of nodes causing equivalent loss intervals in terms of node strength was higher—for instance, 8% for pork and 6.2% for beef.

It is particularly noteworthy that the failure of node strength for specific nodes demonstrated extraordinary disruptive power, such as China (28.7%), the United States (21.5%), Australia (40.1%), and Brazil (29.4%). This further confirms that scale-free networks are robust against random failures but highly vulnerable to targeted attacks.

**Fig 15:**
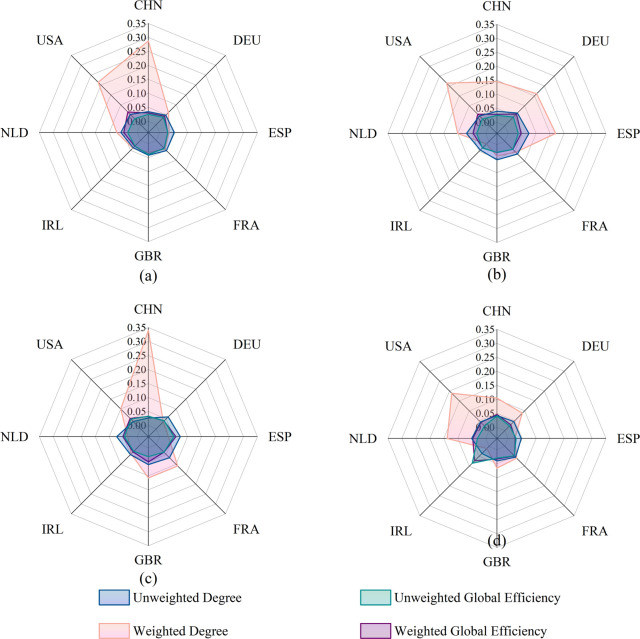
Comparison of performance loss rates in trade networks due to disruptions at critical nodes in 2023.

From Table 6, it can be seen that the degree of disruption caused by the failure of the same node varies across different network performances, with some nodes showing significant differences. To further analyze the impact of core node failures on network performance from multiple perspectives, we constructed a radar chart showing the network performance loss rate under core node failure scenarios, as shown in Fig 15.

**Table 6:** Node importance ranking based on single node disruption network performance loss rate in 2023.

No.	Node	beef	Node	pork	Node	mutton	Node	poultry	Node	beef	Node	pork	Node	mutton	Node	poultry
	Unweighted Global Efficiency Loss Rate								Total Node Degree Loss Rate							
1.000	ARE	0.078	AUT	0.085	ARE	0.153	IRL	0.085	NLD	0.058	ESP	0.074	ESP	0.074	FRA	0.054
2.000	NAM	0.046	NAM	0.063	NZL	0.091	FRA	0.047	ESP	0.053	NLD	0.069	NZL	0.073	NLD	0.050
3.000	FRA	0.041	DNK	0.059	NAM	0.059	TUR	0.046	USA	0.052	FRA	0.064	NLD	0.073	BRA	0.049
4.000	GBR	0.039	POL	0.053	ESP	0.051	CHN	0.041	FRA	0.051	DEU	0.063	FRA	0.067	ESP	0.047
5.000	DEU	0.036	DEU	0.043	USA	0.049	NAM	0.039	ITA	0.049	ITA	0.059	AUS	0.063	DEU	0.046
6.000	NLD	0.034	DMA	0.042	NLD	0.046	GAB	0.038	DEU	0.048	DNK	0.057	DEU	0.060	POL	0.044
7.000	USA	0.030	FRA	0.040	AUS	0.041	DMA	0.033	BRA	0.044	POL	0.055	GBR	0.060	USA	0.043
8.000	AUT	0.030	ESP	0.036	ITA	0.041	GBR	0.030	POL	0.042	GBR	0.054	ITA	0.055	CHN	0.042
9.000	PRY	0.030	IRL	0.034	DEU	0.040	ZAF	0.030	GBR	0.041	BEL	0.052	IRL	0.051	GBR	0.040
10.000	ESP	0.029	ARE	0.034	FRA	0.038	NLD	0.030	AUT	0.041	USA	0.049	ARE	0.047	BEL	0.038
**No.**	**Weighted Global Efficiency Loss Rate**								**Total Node Strength Loss Rate**							
1.000	ARE	0.068	AUT	0.071	ARE	0.145	IRL	0.073	CHN	0.287	USA	0.215	AUS	0.401	BRA	0.294
2.000	USA	0.064	DNK	0.066	NZL	0.106	FRA	0.049	USA	0.214	ESP	0.170	CHN	0.340	USA	0.189
3.000	NLD	0.048	POL	0.063	ESP	0.058	NLD	0.046	BRA	0.186	DEU	0.161	NZL	0.286	NLD	0.138
4.000	DEU	0.042	DEU	0.058	AUS	0.054	CHN	0.046	AUS	0.112	CHN	0.146	FRA	0.108	POL	0.106
5.000	NAM	0.040	USA	0.058	ITA	0.054	TUR	0.044	NLD	0.073	MEX	0.121	GBR	0.108	CHN	0.105
6.000	FRA	0.040	NAM	0.047	USA	0.052	USA	0.041	ARG	0.069	NLD	0.100	USA	0.100	DEU	0.088
7.000	GBR	0.038	ESP	0.047	NLD	0.051	BRA	0.038	IND	0.068	CAN	0.100	ARE	0.053	MEX	0.084
8.000	PRY	0.034	NLD	0.045	GBR	0.050	GAB	0.034	DEU	0.061	ITA	0.089	IRL	0.047	GBR	0.066
9.000	BRA	0.032	FRA	0.040	NAM	0.049	GBR	0.033	CAN	0.061	BRA	0.088	DEU	0.035	FRA	0.060
10.000	AUT	0.030	DMA	0.037	DEU	0.039	NAM	0.033	JPN	0.052	DNK	0.087	NLD	0.034	BEL	0.050

Flow Hubs excel due to their trade volume, with their node strength loss rate significantly higher than their efficiency loss rate. For instance, China in the lamb network (strength loss rate 0.340) and the United States in the beef network (strength loss rate 0.214) would cause a direct and severe blow to the total trade volume upon their failure, yet they are not located on the critical paths within the network topology.

Structural Hubs, on the other hand, derive their importance from their network position, exhibiting an efficiency loss rate notably higher than their strength loss rate. Ireland in the poultry network is the most typical example, where its global efficiency loss rate (0.085) is more than double its strength loss rate. Germany in the beef and pork networks demonstrates a similar pattern. These countries act as crucial “bridges.” Although they do not handle the largest flows, their failure would severely disrupt the connectivity efficiency of the entire network.

#### 3.3.2 Evolution analysis of maximum loss rate in network performance due to single node disruptions.

**Fig 16:**
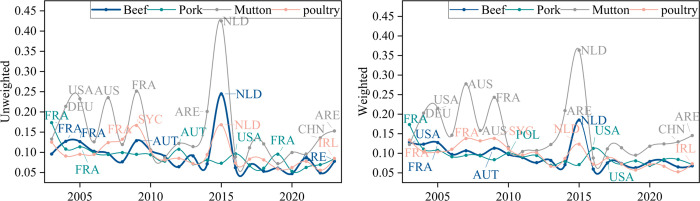
Evolution of the maximum global efficiency loss rate due to a single node of interruption.

**Fig 17:**
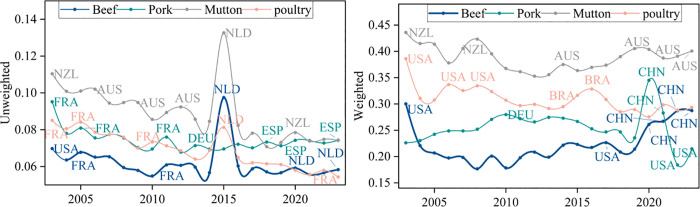
Evolution of the maximum loss rates of node strength due to a single node of interruption.

To analyze the evolution of network vulnerability, we examine the maximum performance loss rate caused by deleting the most vulnerable node each year. This approach reveals the extreme manifestation of vulnerability over time. This approach aims to reveal the extreme manifestation of network vulnerability in the time dimension. The temporal distribution of this indicator is shown in Fig 16, Fig 17, with analysis conducted from both unweighted and weighted perspectives.

As shown in the Fig 16 and Fig 17, there was a significant fluctuation in the maximum loss rate in 2015. The primary reason for this was the failure of the Dutch node, which led to a sudden increase in the performance loss rates of the global frozen beef, pork, and poultry trade networks. Further analysis revealed that, in that year, Dutch established new trade relationships with multiple countries, resulting in a dense increase in trade connections. Subsequently, the network returned to its historical state, and this process caused the fluctuation in the maximum loss rate for that year.

To further analyze the most disruptive nodes to the network each year and their evolutionary process, the nodes causing the greatest disruption each year were further compiled. The statistical data is shown in Table 7.

**Table 7:** The most destructive nodes and their occurrence frequencies from 2003 - 2023.

classification	Beef		Pork		Mutton		Poultry	
	country	frequency	country	frequency	country	frequency	country	frequency
Unweighted global efficiency	FRA	10	FRA	9	ARE	6	FRA	10
	ARE	4	USA	4	FRA	4	CHN	3
Weighted global efficiency	USA	10	FRA	6	ARE	5	FRA	8
	FRA	6	USA	6	USA	5	CHN	5
Node degree	FRA	10	ESP	10	NZL	12	FRA	20
	NLD	10	FRA	8	AUS	6	NLD	1
Node strength	USA	16	DEU	16	NZL	11	BRA	14
	CHN	5	CHN	2	AUS	10	USA	7

#### 3.3.3 Dynamic resilience evolution analysis of trade networks.

**Fig 18:**
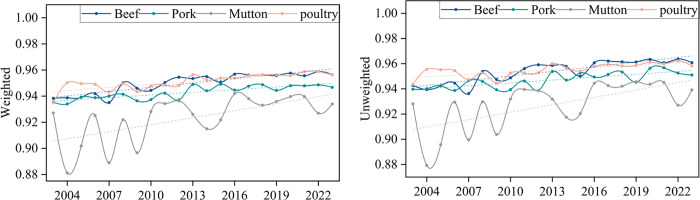
Evolution of the average retention rate of global efficiency due to the interruption of core nodes.

**Fig 19:**
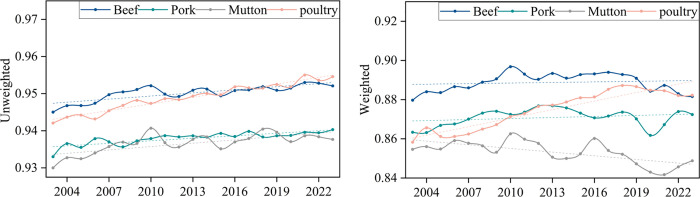
Evolution of the average retention rate of node strength due to the interruption of core nodes.

The evolutionary analysis of network dynamic resilience mainly involves calculating the average loss rate of network performance after the deletion of core nodes each year, in order to assess the contribution of key nodes to the overall stability of the network and their potential risks. The temporal distribution of this metric is shown in Fig 18 and Fig 19, with the analysis conducted from both the unweighted and weighted network dimensions.

From Fig 18 and Fig 19, it is evident that the retention rate of the trade network remains high, whether unweighted or weighted. This indicates that even with the failure of core nodes, the trade network can maintain a relatively high level of efficiency and structural integrity, demonstrating certain resilience. As the network continues to evolve and optimize, the “single node vulnerability” of core nodes decreases, and the network’s invulnerability to the failure of core nodes strengthens. With the increasing diversification of network trade, the network is no longer overly reliant on a few core nodes but instead distributes trade across more nodes, forming more alternative paths and redundant connections. This allows the network to maintain high connectivity even when certain core nodes fail.

Except for mutton, the retention rate trends for different meats are similar, showing an upward trend. This indicates that the network is more adaptable and resilient, with higher substitutability and flexibility in the supply chain, which can better cope with the interruption of core nodes. Comparing different types of meat, the beef network shows the highest resilience, followed by poultry and pork. The mutton trade network experiences a decline in node strength after core node disruptions, mainly due to its high dependence on core nodes and relatively fragile supply chains.

## 4. Discussion and conclusion

This study constructs a global frozen meat trade network from 2003 to 2023, combining complex network theory and dynamic simulation models, revealing its spatiotemporal evolution patterns and structural resilience characteristics. The main conclusions are as follows:

(1) The trade pattern shows a coexisting trend of “multi-polarization” and “regionalization”: while the number of nodes in the global frozen meat trade network has slightly decreased, the number of trade connections and network density have continued to rise, and the trade links between core countries have been continuously deepened. China has firmly established itself as the largest import hub, with beef imports highly dependent on Brazil and Argentina, and pork imports concentrated on Spain and Brazil; Brazil occupies a dominant position in beef and poultry exports. The United States, the Netherlands, the United Arab Emirates and other countries play key hub roles in different categories. Traditional exporting countries (such as Australia and New Zealand) have seen a decline in market share, while emerging hubs (such as the Netherlands and the United Arab Emirates) have significantly improved their status, reflecting the dynamic optimization and regional restructuring of the trade network structure. In terms of trade scale, the global frozen meat trade volume increased from 41.959 billion US dollars to 137.207 billion US dollars, with a compound annual growth rate of 11.4%; the trade quantity rose from 20.349 billion kilograms to 37.468 billion kilograms, with an annual growth rate of 7.0%. Among them, poultry accounts for the highest proportion in trade quantity (>37.8%), while beef, due to its high value-added attribute, accounts for more than 40% of the trade volume, reflecting the differentiated positioning of different meats in global trade.
(2) The resilience of the static structure exhibits hierarchical and disassortative characteristics: The frozen meat trade network has significant scale-free properties, with the node strength distribution following a power-law distribution, and the weighted network showing prominent hierarchy (e.g., the power-law exponents of the pork and beef networks in 2023 were 4.93 and 4.48, respectively). The global efficiency of the network has continued to improve, with the poultry network having the strongest conductivity and the lamb network the weakest, and conductivity is positively correlated with network density. Except for the weighted assortativity coefficients of pork and beef being briefly positive in some years, all other networks show significant disassortativity (negative coefficients), indicating that the network relies on “hub” nodes to connect peripheral countries, forming a “core-periphery” structure.
(3) The resilience of the dynamic structure has been enhanced, and the invulnerability has been gradually improved: Node disruption simulation results show that the failure of core nodes (e.g., China, the United States, Brazil, Australia) exerts a significant impact on the performance of the weighted network. For instance, China’s node strength loss rate in the beef network reaches 28.7%, while Australia’s in the lamb network is as high as 40.1%. However, over time, the network has demonstrated strong resilience when confronting core node failures, with the average retention rates of global efficiency and node strength both exceeding 80%. This indicates that the network has effectively mitigated the risk of single-point dependence through path diversification and redundancy mechanisms. The beef network boasts the strongest dynamic resilience, followed by the poultry and pork networks, whereas the lamb network is the most vulnerable due to its high reliance on a small number of export nodes (e.g., New Zealand, Australia). Time-series analysis further reveals that an abnormal peak in the network performance loss rate occurred in 2015 as a result of the surge in connections of the Dutch node, which further demonstrates that the network structure is constantly adjusting and optimizing in a dynamic manner.
